# Supplementary material for: Fluorogenic RNA Mango aptamers for imaging small non-coding RNAs in mammalian cells
Source: Nat Commun. 2018 Feb 13;9:656. doi: 10.1038/s41467-018-02993-8 (PMC5811451; doi:10.1038/s41467-018-02993-8)
Supplement: Supplementary file 1 — Supplementary Information [file 41467_2018_2993_MOESM1_ESM.pdf]

TO1-Biotin

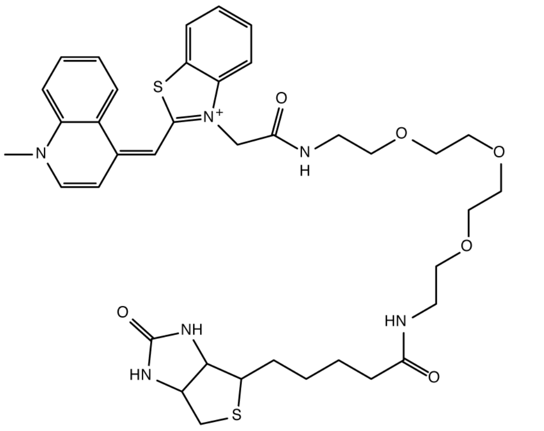

TO3-Biotin

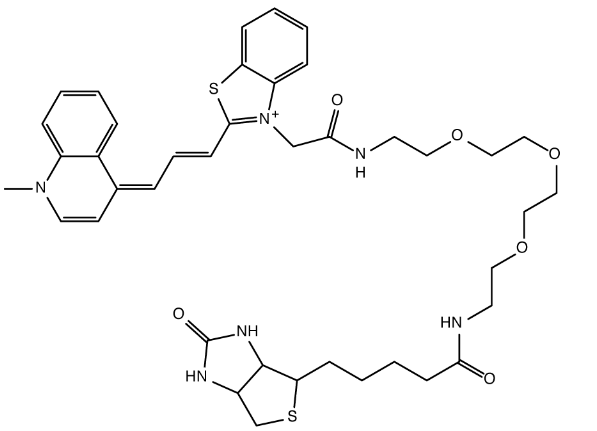

N-methyl mesoporphyrin IX
(NMM)

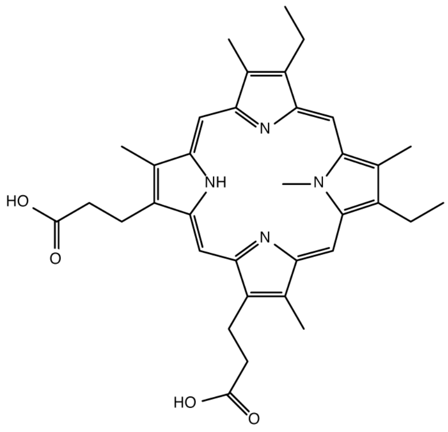

Supplementary Figure 1 | Chemical structures of TO1-Biotin, TO3-Biotin (ex. co. 133,000 $M^{-1}cm^{-1}$ @ 615 nm), and NMM.

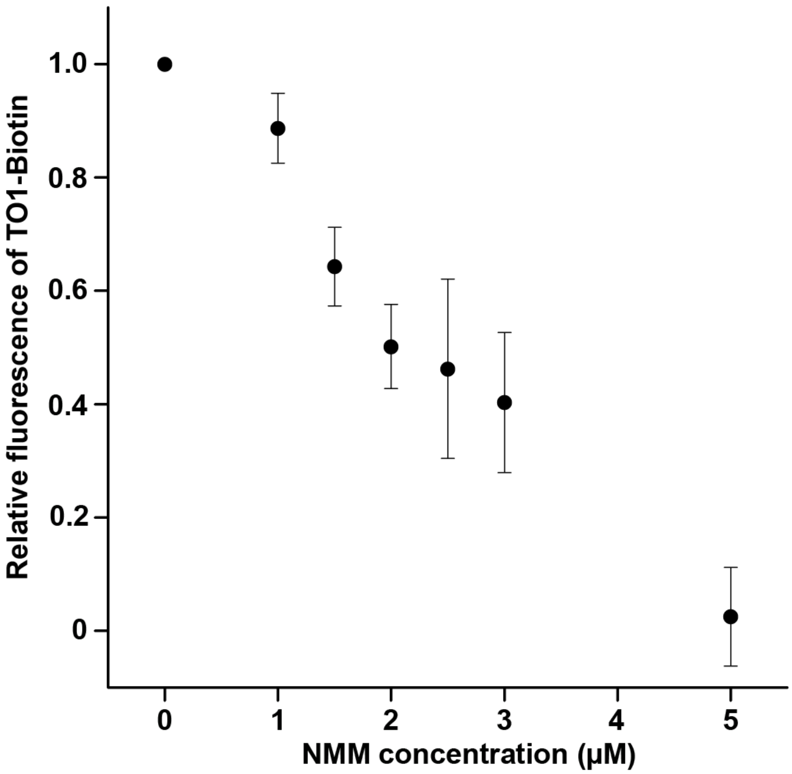

Supplementary Figure 2 | Effect of N-methyl mesoporphyrin IX (NMM) concentration on TO1-biotin/Mango I fluorescence. RNA aptamer at 1 µM was incubated with 100 nM of TO1-Biotin and a concentration of NMM ranging from 0 to 5 µM. Green fluorescence was recorded (ex: 492 nm/em: 516 nm) in a real-time thermocycler (Mx 3005P, Agilent). Values are normalized to that of the 0 µM NMM point and are the mean of two independent experiments and error bars correspond to ± 1 standard error.

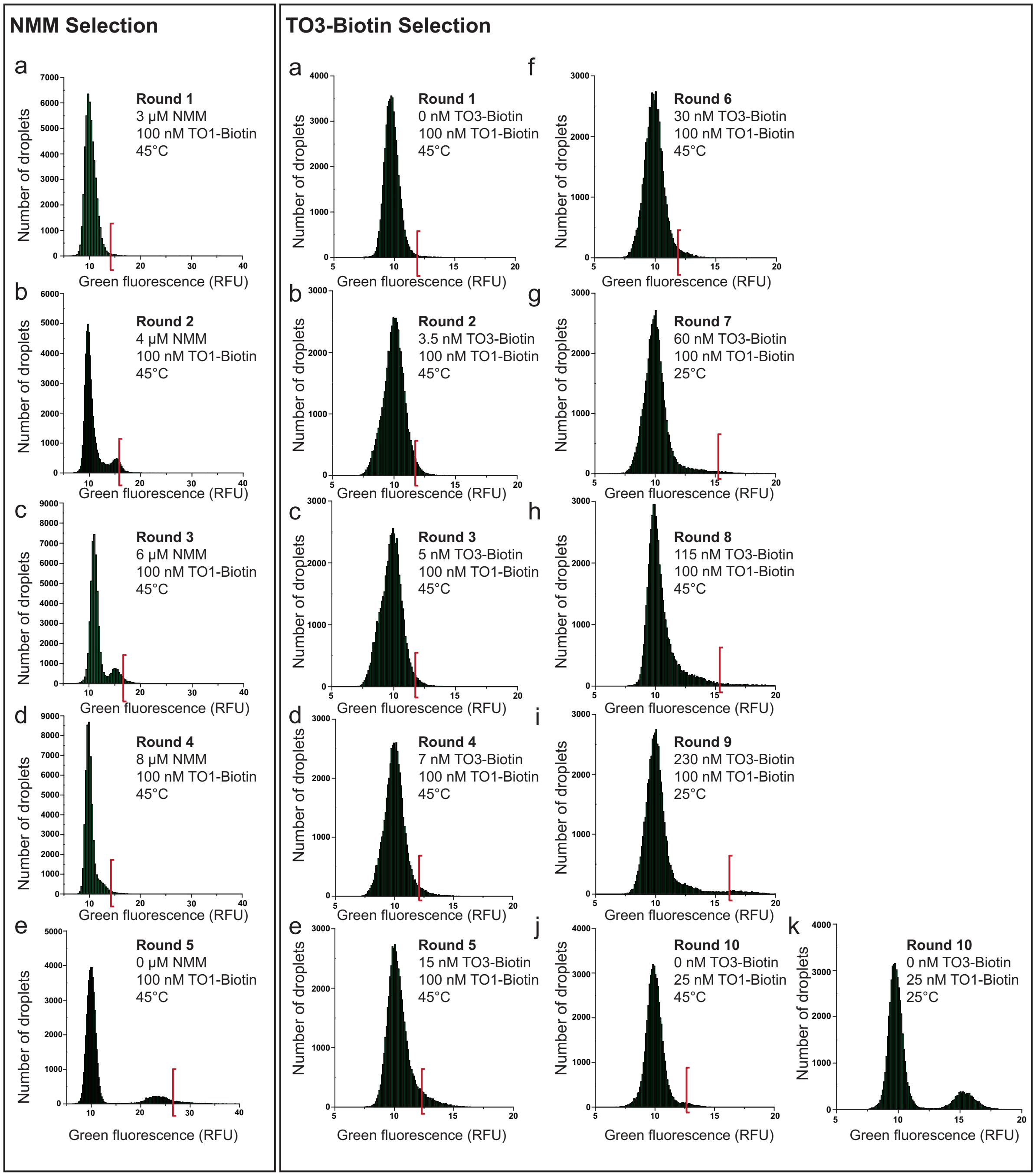

Supplementary Figure 3 | Green fluorescence profile of the screenings performed in the presence of NMM (Rounds 1 to 5 left panel panels a to e) or TO3-Biotin (Rounds 1 to 10 right panels a to j). The green fluorescence of 50,000 droplets was used to build each profile. Red bars indicate the limit over which droplets were gated and sorted as positive. The selection conditions (TO1-Biotin and NMM/TO3-Biotin concentrations as well as the temperature of the analysis device) are indicated. In the TO3-Biotin screening, the two last experiments (**j** and **k**) correspond to the same experiment performed respectively at 45°C (**j**) and 25 °C (**k**).

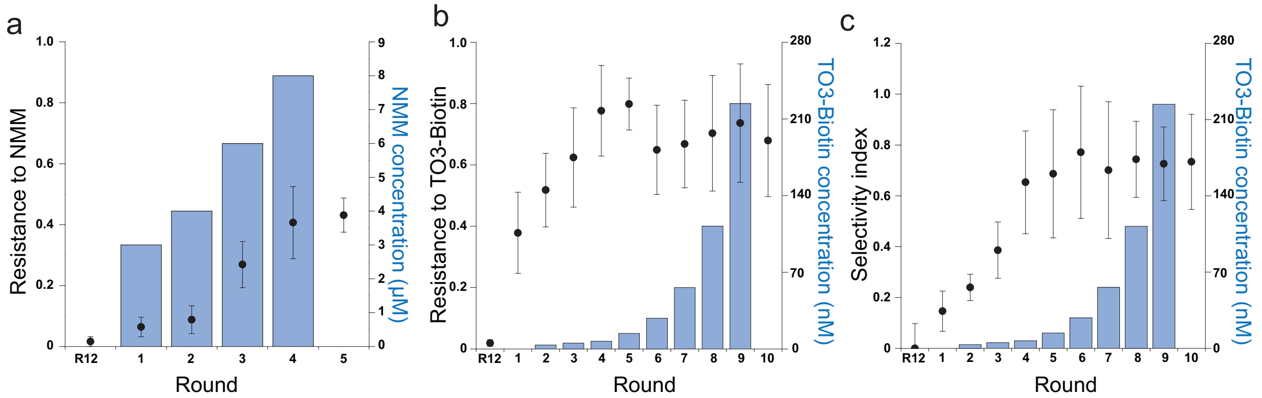

Supplementary Figure 4 | Resistance or selectivity of TO1-Biotin binding variants in presence of competitors after each round of screening. (a) NMM resistance selection: Resistance of TO1-Biotin/RNA complexes to NMM. The fluorescence of the complex between TO1-Biotin and the RNAs from the libraries obtained after each round of screening was determined by mixing 2 µM RNA and 100 nM TO1-Biotin in the absence or in the presence of 3 µM NMM. The Resistance to NMM was calculated by normalizing the aptamer/TO1-Biotin fluorescence in the presence of NMM by the aptamer/TO1-Biotin fluorescence in the absence of NMM. **(b) TO3-Biotin resistance selection:** Resistance of TO1-Biotin/RNA complexes to TO3-Biotin. The fluorescence of the complex between TO1-Biotin and the RNAs from the libraries obtained after each round of screening was determined by mixing 300 nM RNA and 100 nM TO1-Biotin, in the absence or in the presence of 110 nM TO3-Biotin. The resistance to TO3-Biotin was calculated by normalizing the aptamer/TO1-Biotin fluorescence in the presence of TO3-Biotin by the aptamer/TO1-Biotin fluorescence in the absence of TO3-Biotin. **(c)** Selectivity of the libraries obtained after the different rounds of screening in the presence of TO3-Biotin. The green fluorescence of the TO1-Biotin/RNA complex was normalized to the red fluorescence of the TO3-Biotin/RNA complex to calculate the selectivity index. The blue bar indicates the concentration of competitor used during the screening step. Data was obtained at 25 °C, the values are the mean of three independent experiments and error bars correspond to ± 1 standard error.

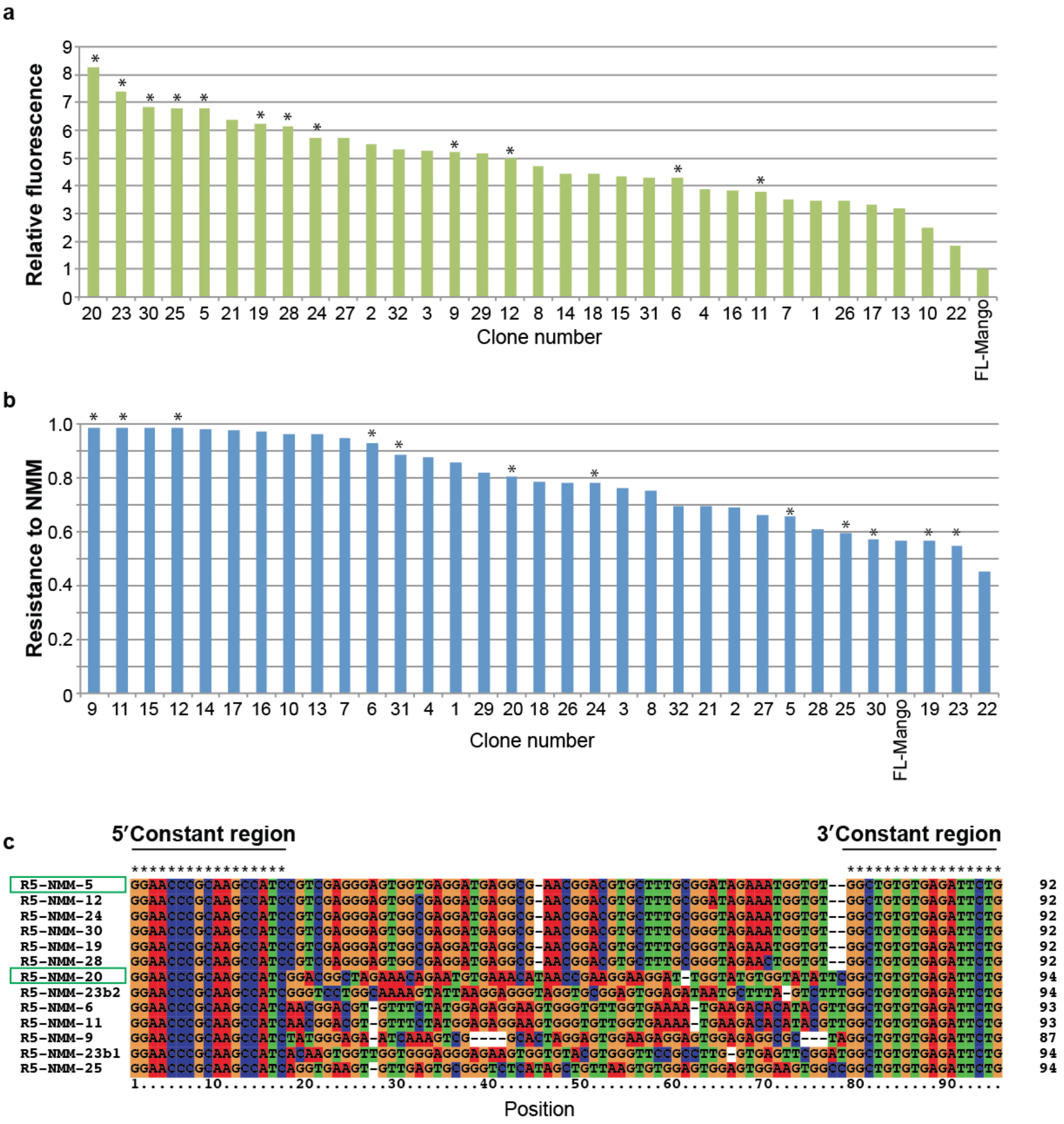

Supplementary Figure 5 | Analysis of the clones obtained at the end of the screening process performed in the presence of NMM. (a) Brightness of the complexes formed between TO1-Biotin and individual variants isolated from the screenings in the presence of NMM. Aptamer-coding genes were PCR amplified, *in vitro* transcribed in the presence of TO1-Biotin and the fluorescence was monitored at 37 °C. The maximal fluorescence was normalized to that of Mango I. (**b)** Resistance of TO1-Biotin/RNA complex to NMM. TO1-Biotin fluorescence was monitored as in (**a)** in the absence or in the presence of 3 µM NMM. **(c)** Sequence analysis of the clones of interest. The sequences of the clones of interest (indicated by an asterisk in **a** and **b**) were aligned with Clustal X. The green boxes indicate Mango III (R5-NMM-20) and Mango IV (R5-NMM-5).

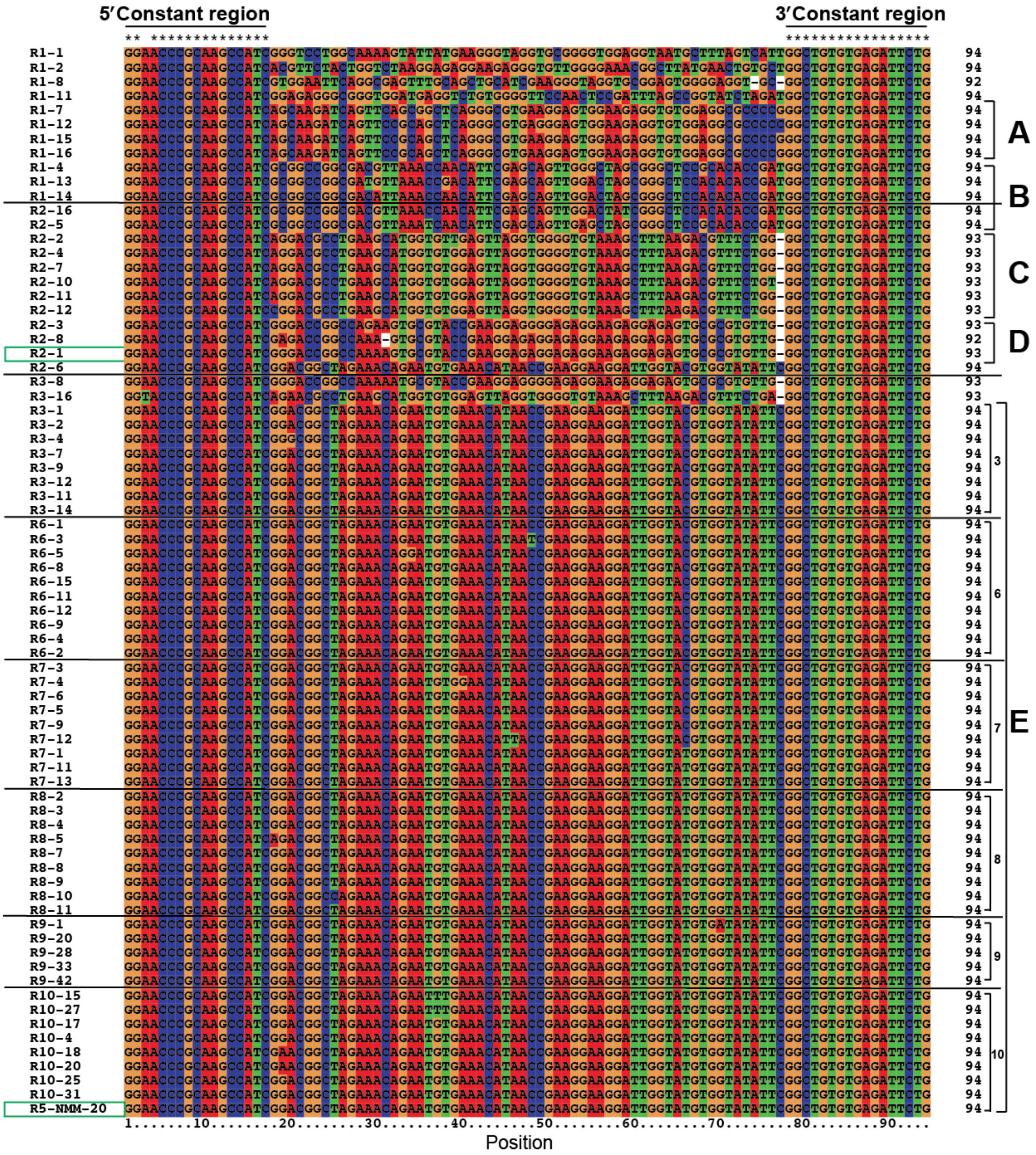

Supplementary Figure 6: Sequence analysis of the variants isolated across the rounds of screening performed in the presence of TO3-Biotin.

The first green box indicates Mango II (R2-1). For comparison, the sequence of Mango III (R5-NMM-20) was also added and boxed in green. The families of sequences clustering together are labelled (A to E).

a

Construct	Sequence	Rel. F_E	K_D
R2-1 (full-length)	...GTGCGTACC-GAA-GG-AGA-GG-AGA-GG-AAGA-GG-AGA-GTGCGCGTGTTG...	1.0 ± 0.2	0.6 ± 0.1
Mango II	GGCACGTAC-GAA-GG-AGA-GG-AGA-GG-AAGA-GG-AGA-GTACGTGC	1.0 ± 0.2	0.3 ± 0.1
Variant 1	GGCACGTAC-GAA-GG-AGA-GG-TGC-GG--AGA-GG-AGA-GTACGTGC	0.9 ± 0.2	3.5 ± 0.3
Variant 2	GGCACGTAC-GAA-GG-GAC-GG-AGA-GG--AGA-GG-AGA-GTACGTGC	0.7 ± 0.1	7.1 ± 0.4
Variant 3	GGCACGTAC-GAA-GG-GAC-GG-TGC-GG-AAGA-GG-AGA-GTACGTGC	1.1 ± 0.2	8.2 ± 1.7
Variant 4	GGCACGTAC-GAA-GG-AGA-GG-AGA-GG--AGA-GG-AGA-GTACGTGC	0.7 ± 0.4	4.7 ± 0.8
Variant 5	GGCACGTAC-GAA-GG-AGA-GG-TGC-GG-AAGA-GG-AGA-GTACGTGC	0.9 ± 0.2	0.9 ± 0.1
Variant 6	GGCACGTAC-GAA-GG-GAC-GG-AGA-GG-AAGA-GG-AGA-GTACGTGC	1.1 ± 0.1	12 ± 1
Variant 7	GGCACGTAC-GAA-GG-AGA-GG-AGA-GG-ATGA-GG-AGA-GTACGTGC	0.9 ± 0.2	2.5 ± 0.3

b

Construct	Sequence	Rel. F_E	K_D
R5-NMM-5 (full-length)	...ACCCGCAAGCCATCCGT-CGA-GG-GAGT-GG-TGA-GG-ATGA-GG-CGA-ACGGACGTGCTTTG...	1.0 ± 0.2	10 ± 1
Mango IV	--------GGCACGTAC-CGA-GG-GAGT-GG-TGA-GG-ATGA-GG-CGA-GTACGTGC	1.0 ± 0.2	13 ± 1
Variant 1	---------------------GG-GAGT-GG-TGA-GG-ATGA-GG-CGA-ACGGACGTGCTTTG...	0.3 ± 0.1	75 ± 5
Variant 2	--GGCGGACGTGCTTTG...	u.d.	u.d.
Variant 3	GGCCGCAAGCCATCCGT-CGA-GG-GAGT-GG-TGA-GG-ATGA-GG-CGA-ACGGACGTGCTTTG...	0.9 ± 0.1	28 ± 2
Variant 4	-----GGAAGCCTCCGT-CGA-GG-GAGT-GG-TGA-GG-ATGA-GG-CGA-ACGGAGTGCTT	0.9 ± 0.2	7 ± 1
Variant 5	--------GGACGTACT-CGA-GG-GAGT-GG-TGA-GG-ATGA-GG-CGA-AGTACGTC	1.0 ± 0.2	7 ± 2
Variant 6	--------GGCACGTA--CGA-GG-GAGT-GG-TGA-GG-ATGA-GG-CGA-GTACGTGC	0.5 ± 0.1	18 ± 10
Variant 7	--------GGCACGTA--CAG-GG--AGT-GG-TGA-GG-ATGA-GG-CG--GTACGTGC	0.8 ± 0.2	10 ± 3
Variant 8	--------GGCACGTAC-GAA-GG-GAGT-GG-TGA-GG-ATGA-GG-AGA-GTACGTGC	0.6 ± 0.1	20 ± 1
Variant 9	--------GGCACGTAC-CGA-GG-GAC--GG-TGA-GG-ATGA-GG-CGA-GTACGTGC	0.2 ± 0.1	250 ± 20
Variant 10	--------GGCACGTAC-CGA-GG-GAGT-GG-TGC-GG-ATGA-GG-CGA-GTACGTGC	0.9 ± 0.2	10 ± 1
Variant 11	--------GGCACGTAC-CGA-GG-GAGT-GG-TGA-GG-A-GA-GG-CGA-GTACGTGC	0.5 ± 0.1	7 ± 1
Variant 12	--------GGCACGTAC-CGA-GG-AAGT-GG-TGA-GG-ATGA-GG-CGA-GTACGTGC	0.4 ± 0.1	70 ± 20
Variant 13	--------GGCACGTAC-CGA-GG--AGT-GG-TGA-GG-ATGA-GG-CGA-GTACGTGC	0.4 ± 0.1	6 ± 1
Variant 14	--------GGCACGTAC-CGA-GG-GAAT-GG-TGA-GG-ATGA-GG-CGA-GTACGTGC	0.3 ± 0.1	300 ± 30
Variant 15	--------GGCACGTAC-CGA-GG-GA-T-GG-TGA-GG-ATGA-GG-CGA-GTACGTGC	0.2 ± 0.1	140 ± 20
Variant 16	--------GGCACGTAC-CGA-GG--AGA-GG-TGA-GG-ATGA-GG-CGA-GTACGTGC	0.7 ± 0.2	7 ± 1
Variant 17	--------GGCACGTAC-CGA-GG-GAGT-GG-TGA-GG-AAGA-GG-CGA-GTACGTGC	0.6 ± 0.1	40 ± 2
Variant 18	--------GGCACGTAC-CGA-GG-GAGT-GG-TGA-GG-ATGA-GG-AGA-GTACGTGC	0.6 ± 0.1	26 ± 1
Variant 19	--------GGCACGTAC-CGA-GG-GAGT-GG-TGA-GG-ATGA-GG-CAA-GTACGTGC	0.8 ± 0.2	25 ± 4
Variant 20	--------GGCACGTAC-GAA-GG-GAGT-GG-TGA-GG-ATGA-GG-CGA-GTACGTGC	0.7 ± 0.1	40 ± 2

c

Construct	Sequence	Rel. F_E	K_D
R5-NMM-20 (full-length)	...GAAACATAACCGAA-GG-AA-GG-ATT-GG-TATGT-GG-TATA-TTCGGCTGTGTGAGA...	1.0 ± 0.1	14 ± 1
Mango III	--GGCACGTACGAA-GG-AA-GG-ATT-GG-TATGT-GG-TATA-TTCGTACGTGCC	1.0 ± 0.1	7.3 ± 0.4
Variant 1	...GAAACATAACCGAA-GG-AA-GG-ATT-GG-TATGT-GG-TATA-TTCGGCTGTGTGAGA...	0.9 ± 0.1	35 ± 2
Variant 2	...GAAACATAACCGAA-GG-AA-GG-ATT-GG-TATGT-GG-TATA-TTCGGCTGTGTG	0.8 ± 0.1	140 ± 8
Variant 3	...GAAACATAACCGAA-GG-AA-GG-ATT-GG-TATGT-GG-TATA-TTCGGCTGTGTGAGA...	0.9 ± 0.1	170 ± 6
Variant 4	--------GGCGAA-GG-AA-GG-ATT-GG-TATGT-GG-TATA-TTCGGCTGTGTGAGA...	1.0 ± 0.1	15 ± 1
Variant 5	...GAAACATAACCGAA-GG-AA-GG-ATT-GG-TATGT-GG-TATA-TTCGGCTGTGTGAGA...	1.0 ± 0.1	12 ± 1
Variant 6	...GAAACATAACCGAA-GG-AA-GG-ATT-GG-TATGT-GG-TATA-TTCGGCTGTGTG	1.1 ± 0.1	52 ± 3
Variant 7	-GGACATAACCGAA-GG-AA-GG-ATT-GG-TATGT-GG-TATA-TTCGGCTGTGTGAGA...	1.1 ± 0.2	12 ± 1
Variant 8	-----GGCACGGAA-GG-AA-GG-ATT-GG-TATGT-GG-TATA-TTCCGTGCC	1.0 ± 0.1	5.6 ± 0.3
Variant 9	-----GGCACGTAC-GG-AA-GG-ATT-GG-TATGT-GG-TATA-GTACGTGCC	1.0 ± 0.1	46 ± 3
Variant 10	-----GGCACGGAA-GG-AA-GG-ATT-GG-TATGT-GG-TATA----CGTGCC	u.d.	u.d.
Variant 11	-----GGCACG----GG-AA-GG-ATT-GG-TATGT-GG-TATA-TTCCGTGCC	u.d.	u.d.
Variant 12	--GGCACGTACGAA-GG-AA-GG-ATT-GG-TACGT-GG-TATA-TTCGTACGTGCC	0.6 ± 0.1	13 ± 1

Supplementary Figure 7: Mutations and truncations of Mangos II, III and IV.
(a) Mango II constructs. **(b)** Mango IV constructs. **(c)** Mango III constructs. F_E is relative to the full-length construct, which was normalized to one. Constructs with binding affinities higher than the end-point of titration are labelled 'u.d.' (undeterminable). The closing stem regions are highlighted in purple. Guanine residues protected from DMS cleavage in the named Mango constructs of the study (**Fig. 3c**) are highlighted in yellow.

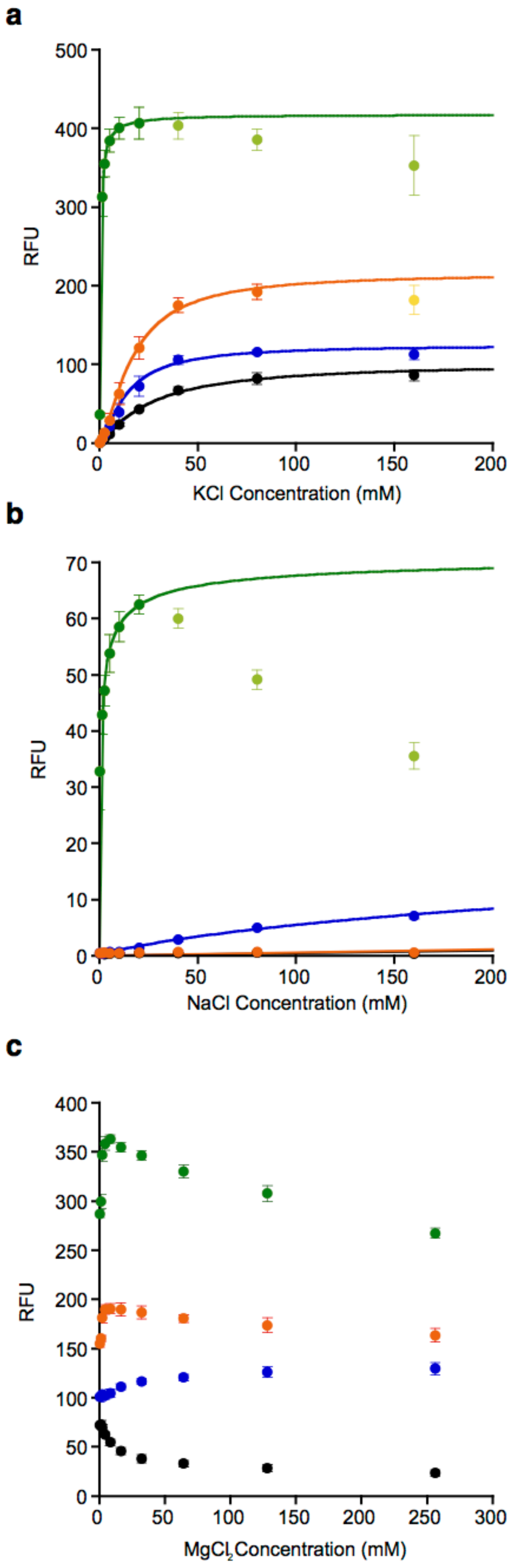

Supplementary Figure 8: Salt dependence of new Mango variants.
Dependence of fluorescence of each Mango for **(a)** K^+, **(b)** Na^+ in place of K^+, and **(c)** Mg^{2+} ions in a buffer containing 140 mM K^+. Each salt was titrated holding 25 nM RNA and 50 nM TO1-Biotin constant. 10 mM Tris buffer (pH 7.2) was used in place of phosphate of the WB buffer to avoid monovalent counter ions. Color-coding is as follows: Black – Mango I, Blue – Mango II, Green – Mango III, Orange – Mango IV. When possible, data is fitted to the Hill equation and Hill coefficients are listed in **Supplementary Table 4**. Hill coefficients are fitted to dark points. The detailed source of this inhibition in fluorescence has not been characterized. Points in lighter shade have been excluded from the fit. Error bars are standard deviations of three replicates.

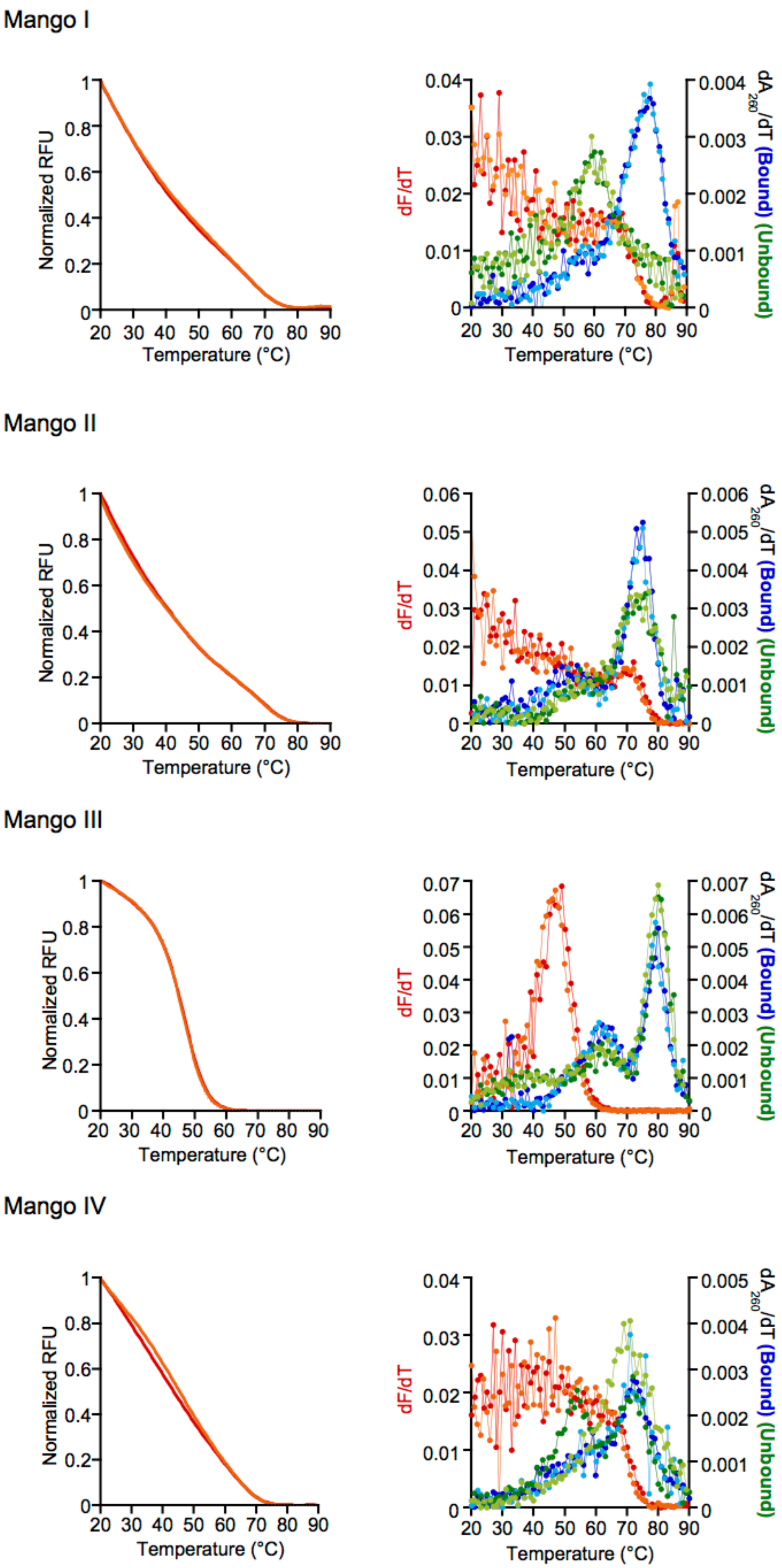

Supplementary Figure 9: Temperature-dependent fluorescent and UV absorbance spectroscopy of new Mango variants. Line plots of temperature-dependent spectroscopy for Mango I (data obtained from previous work)[1], Mango II, Mango III, and Mango IV. Left panels: 1 µM RNA was incubated with 5 µM TO1-Biotin and subjected to temperature ramps while monitoring fluorescence (red shades). Right panels: A_{260} for this sample with 5 µM TO1-Biotin (green shades) and without TO1-Biotin (blue shades) were collected and the simple derivative plotted together with the derivative of the fluorescence data. Starting at 90 °C, temperature was ramped down at a rate of 1 °C/min to 20 °C (darker shade) and returned to 90 °C at a rate of 1 °C/min (lighter shade).

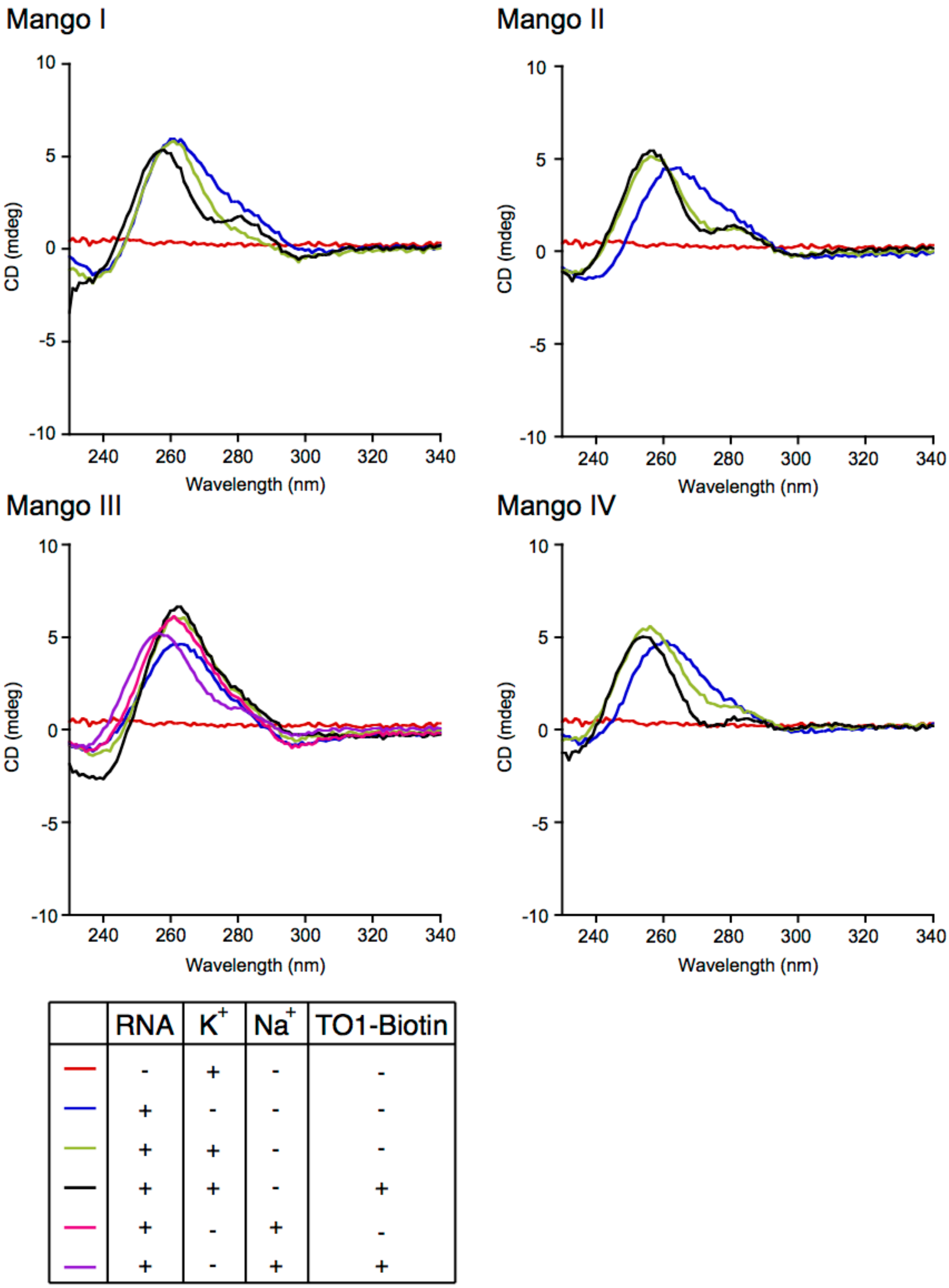

Supplementary Figure 10 | Circular dichroism spectra. Circular dichroism spectra of Mango I, II, III, and IV. 5 µM RNA was measured in 10 mM Tris pH 7.5 buffer either alone, with 140 mM monovalent salt, and/or with 7 µM TO1-Biotin as indicated by the legend. Data is a line plot to guide the eye.

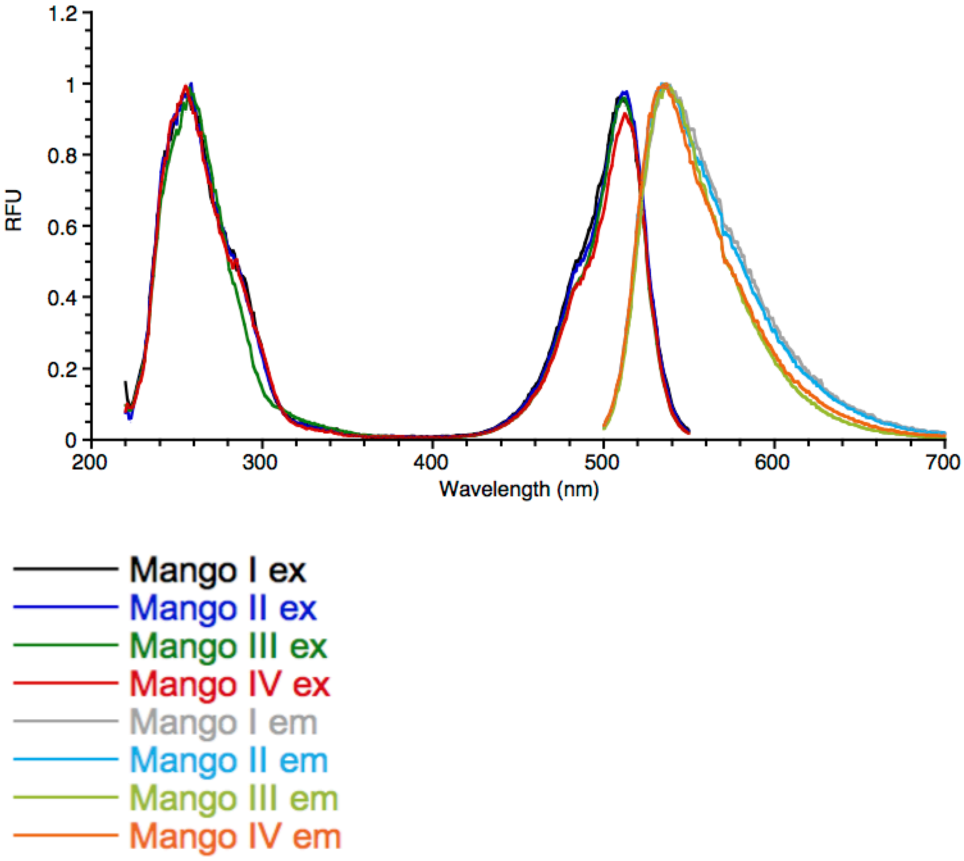

Supplementary Figure 11 | Excitation/emission of the new Mangos. Excitation (dark curve) and emission (light curve) spectra of each Mango. All Mangos have λ_{ex} max = 510 nm and λ_{em} max = 535 nm. Color-coding is as follows: Black – Mango I, Blue – Mango II, Green – Mango III, Red – Mango IV.

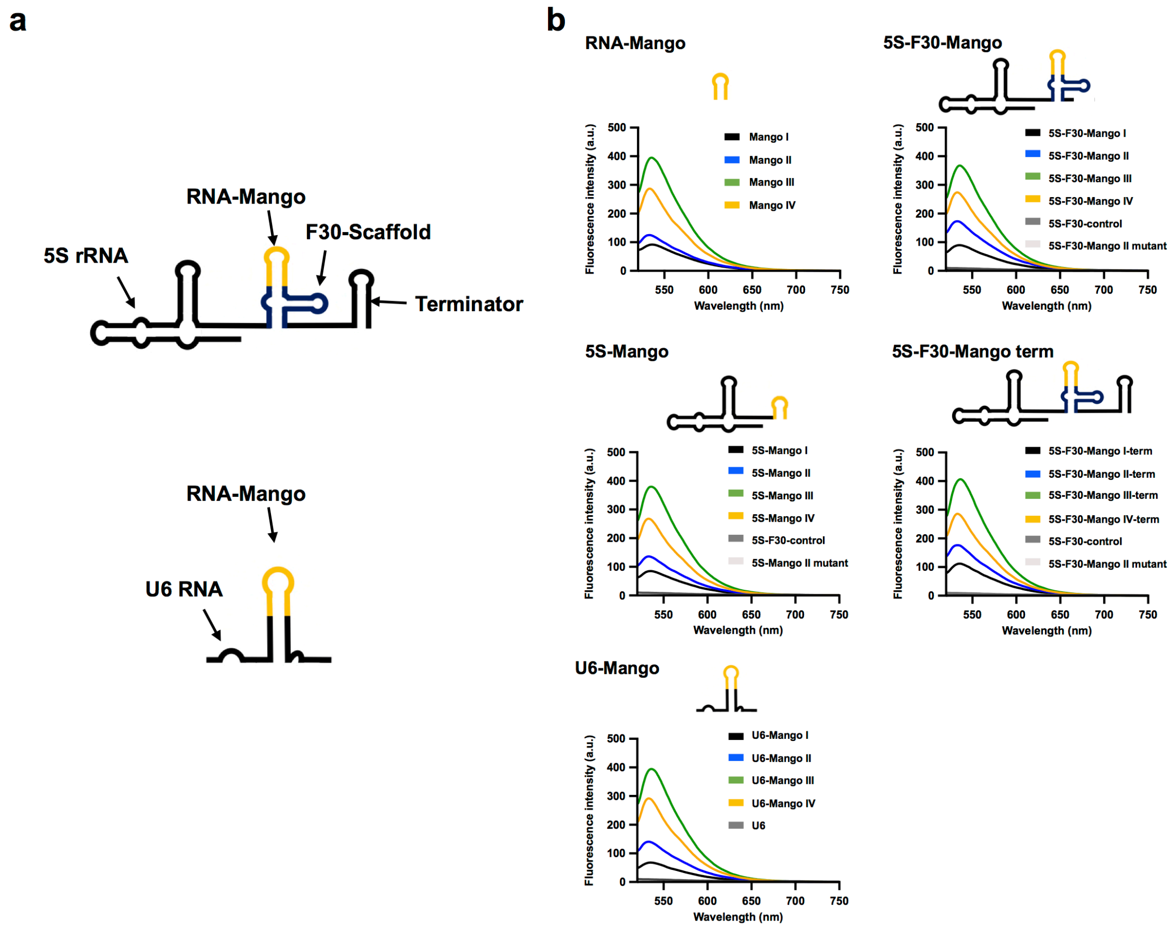

Supplementary Figure 12 | RNA scaffold diagrams. (**a**) Schematic diagrams of the 5S-Mango and U6-Mango constructs synthesized (sequences in **Supplementary Table 5**) and (**b**) their fluorescence emission as a function of wavelength after excitation at 505 nm, compared to unmodified Mango I-IV.

a

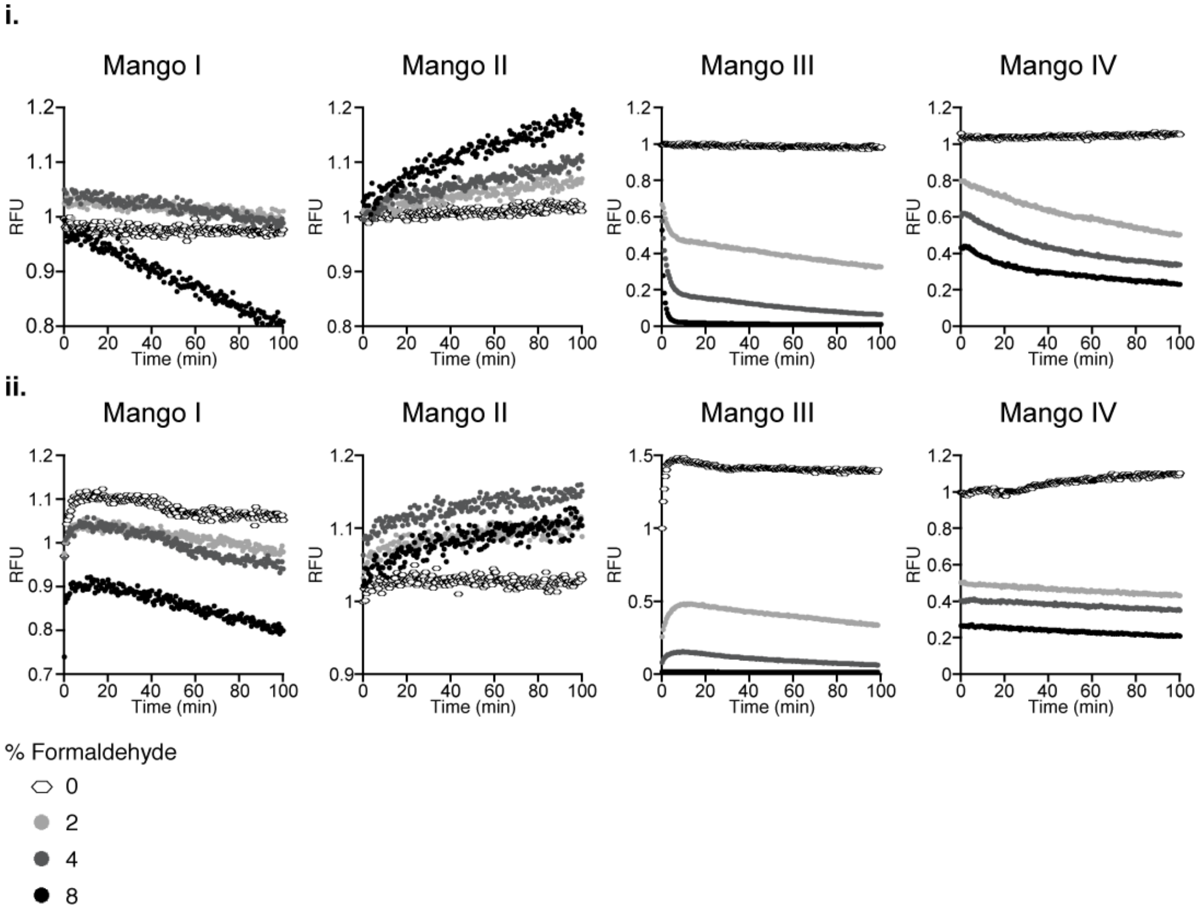

b

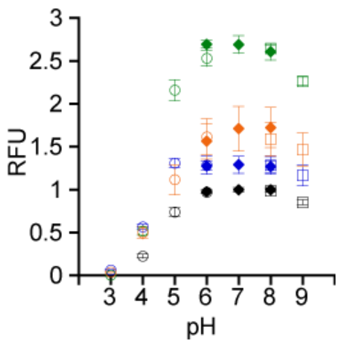

Supplementary Figure 13 | Effect of formaldehyde and pH on Mango fluorescence. a) Fluorescence of 50 nM RNA Mango I, II, III, and IV with 100 nM TO1-Biotin are measured at 30 second intervals at 25 °C. In **i)** RNA is first incubated with TO1-Biotin for one hour before addition of corresponding formaldehyde amounts and fluorescence is measured for another 100 minutes. In **ii)** RNA is incubated with the corresponding amounts of formaldehyde first for one hour, then TO1-Biotin was added. Data for each panel is normalized to RFU at the 0 % formaldehyde, 0 min point. **b)** pH titration for 50 nM Mango (Mango I – black, Mango II – blue, Mango III – green, Mango IV – orange) and 100 nM TO1-Biotin. The fluorescence of each Mango with TO1-Biotin was measured as a function of pH. 50 mM Sodium Citrate buffer for pH 3-6 (open circles), 50 mM Sodium Phosphate for pH 6-8 (filled diamonds, 50 mM Tris for pH 8-9 (open squares). RFU is normalized to 1 for Mango I at pH 7. Errors are standard deviations of 3 replicates.

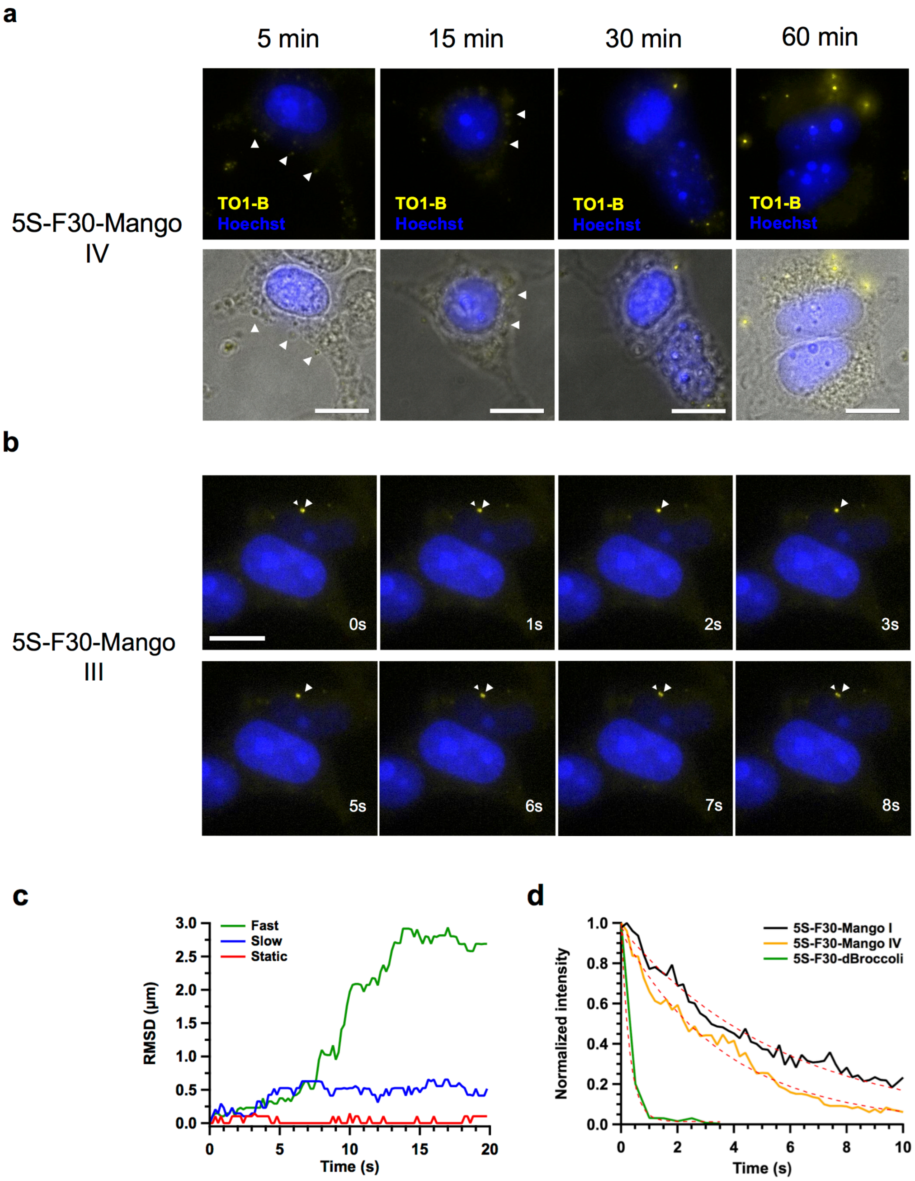

Supplementary Figure 14 | Formation of 5S-Mango foci and their dynamics in live cells. **(a)** 5S-Mango IV imaged post cell fixation at 5, 15, 30 and 60 min after transfection using Lipofectamine based CRISPRMAX transfection reagent. **(b)** Live-cell imaging of 5S-Mango III 60 min post transfection and the RNA (125nM) was pre-incubated with TO1-Biotin (250 nM) prior to transfection. **(c)** Root mean-squared displacement (RMSD) of three observed foci with different diffusive behaviors, fast (Green), slow (Blue) and static (Red) shown in **Supplementary Video 3**. Scale bars are 10 µm and arrows indicate foci of interest. **(d)** Normalized photobleaching traces of 5S-F30-Mango I and IV compared to 5S-F30-dBroccoli (20 µM DFHBI-1T) under constant illumination with an exposure time of 200 ms. A single exponential fit yields the photobleaching half-lives: 5.7 ± 0.2 s (Mango I), 3.7 ± 0.1 s (Mango IV) and 0.30 ± 0.01 s (dBroccoli).

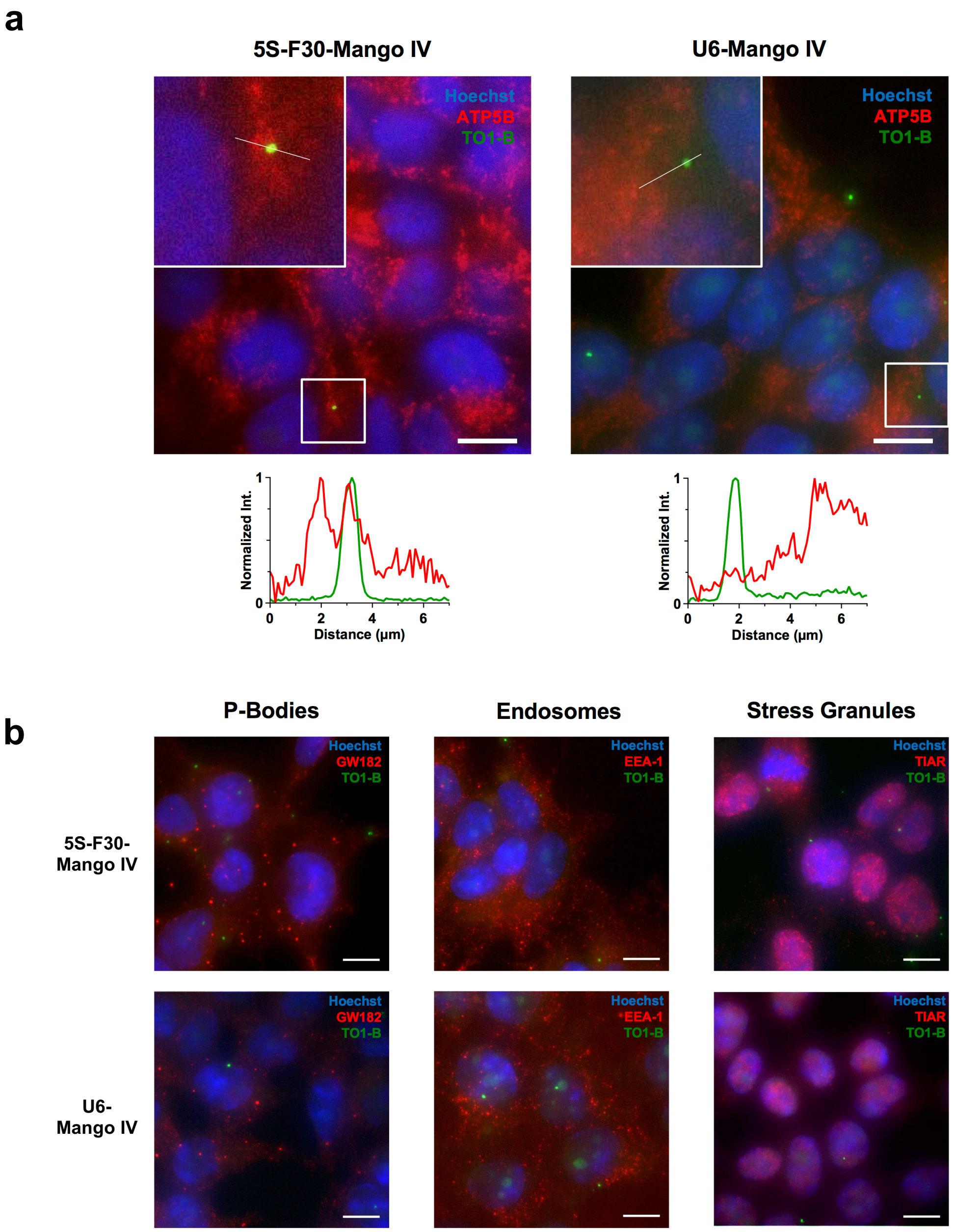

Supplementary Figure 15 | Co-Immunofluorescence with direct RNA-Mango transfections
(a) Cytoplasmic 5S-Mango IV foci are observed to localize with immunostained mitochondria, whereas cytoplasmic U6-Mango foci do not. Cells fixed and stained with TO1-Biotin (200nM – Green), Mitochondrial antibody (ATP5B – Red) and Hoechst 33258 (1µg/ml – Blue). Normalized fluorescence intensities as a function of distance along the inset white lines (bottom panels). **(b)** Localization of 5S-Mango IV and U6-Mango IV foci relative to immunostained P-Bodies (GW182), Endosomes (EEA-1) and Stress Granules (TIAR). Scale bars are 10 µm. All images are maximum projections except in **a**, which show a single focal plane.

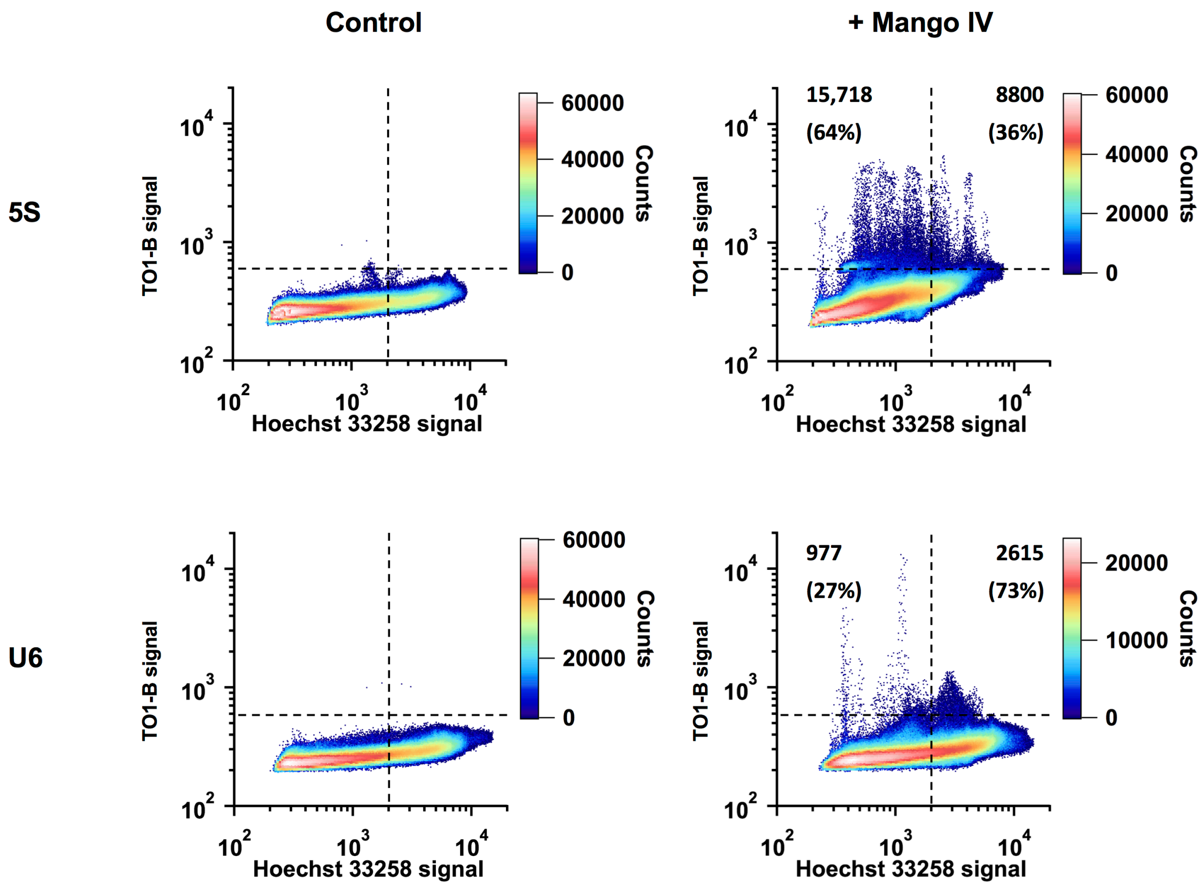

Supplementary Figure 16 | 2D nuclear co-localization plots. Pixel-by-pixel intensity plots of the TO1-Biotin signal (200 nM) vs Hoechst 33258 signal (1 µg/ml) for both 5S-Mango IV and U6-Mango IV compare to their respective controls in fixed cells. For the Mango specific signal, a threshold was set above the 5S-F30-Control background of 600 a.u. Whereas the threshold of the nuclear boundary is observed to be ~2000 a.u. based on Hoechst 33258 staining. The upper quadrants highlight the number of pixels contain within and therefore depict pixels observed outside (upper left) or inside the nucleus (upper right). Each plot contained five maximum projection images with dimensions 1280x1280. The number of cells for 5S-F30-Control, 5S-F30-Mango IV, U6-Control and U6-Mango IV were 57, 114, 131 and 183, respectively.

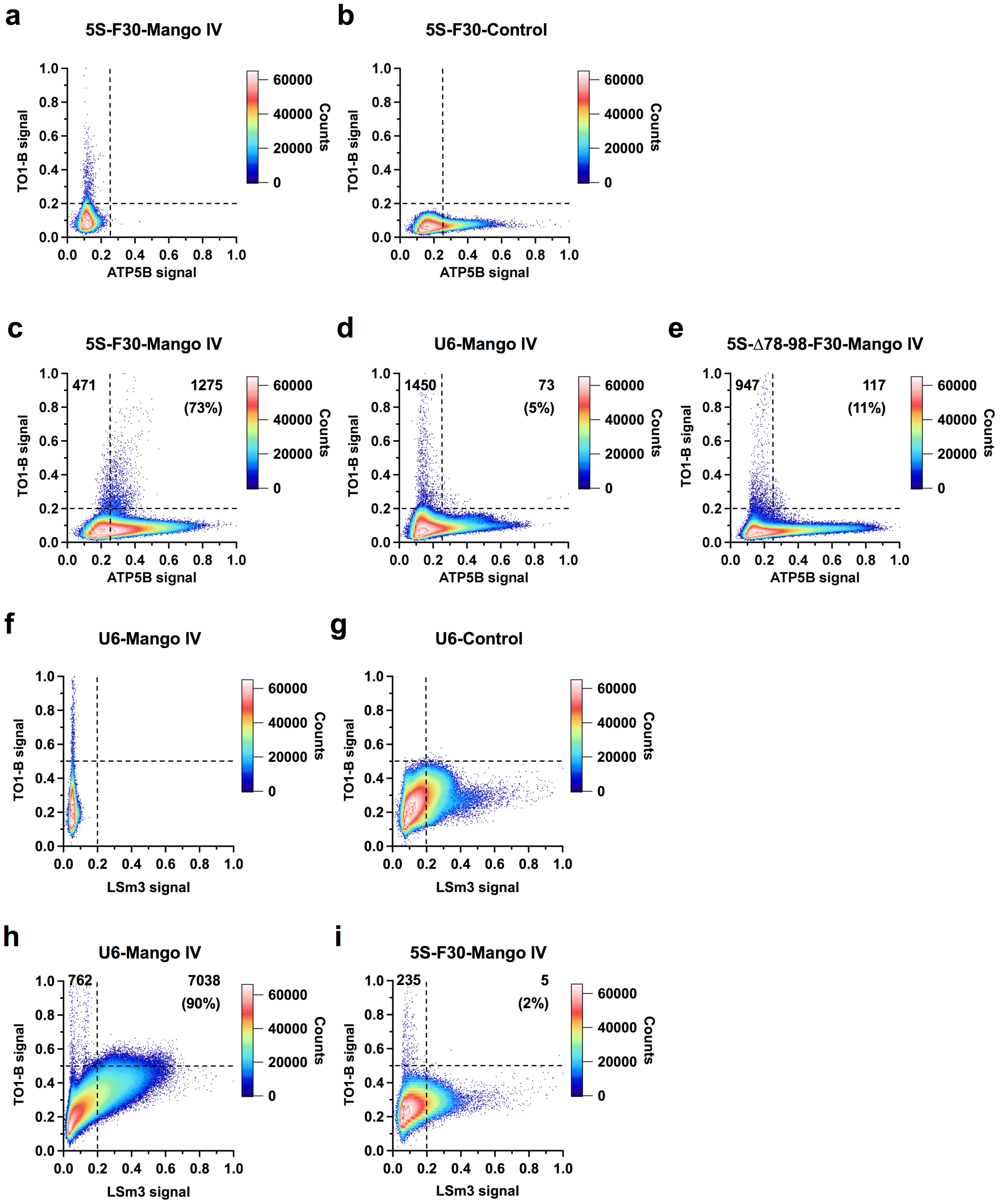

Supplementary Figure 17 | 2D immunostaining co-localization plots. Normalized pixel-by-pixel intensity plots of the TO1-Biotin signal vs. immunostaining signal for both Mitochondria (ATP5B, **a-e**) and snRNPs (LSm3, **f-i**). Background intensity values were determined either with a Mango IV-tagged construct in the absence of immunostaining or a control construct in the presence of both TO1-Biotin and the appropriate immunostain **(a, b, f and g)**. All plots were normalized by subtracting the background signal in each channel and then normalizing to the highest significant pixel intensity from each of the channels. **(c-e)** Show the co-localization patterns of Mango tagged 5S, U6 and 5S Δ78-98 with Mitochondria. **(h and i)** Show co-localization patterns of Mango tagged U6 and 5S with snRNPs. The upper quadrants highlight the number of pixels contained within and therefore depict the co-localized (upper right) and distinct (upper left) Mango signal. Each plot contains multiple slices taken from ~five images with dimensions 1280x1280 to accurately determine co-localizing pixels. The number of cells for plots **a**, **b**, **c**, **d**

and **e** were 66, 26, 178, 217 and 156 respectively. The number of cells for plots **f**, **g**, **h** and **i** were 34, 108, 165, and 89, respectively.

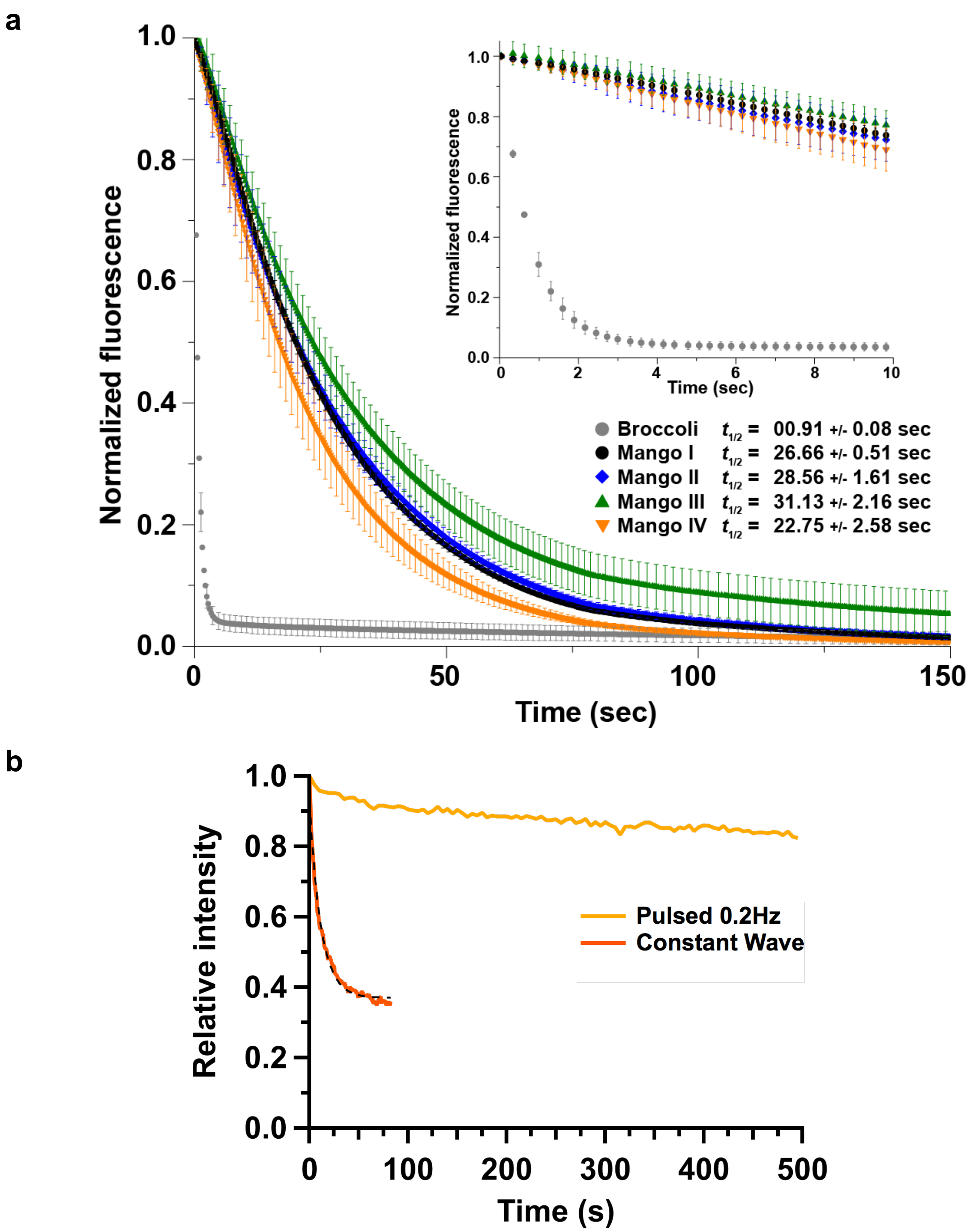

Supplementary Figure 18 | Photostability of new Mango variants *in vitro* and in fixed cells. (**a**) *In vitro* transcribed and purified 5S-F30-Broccoli or 5S-F30-Mango I to IV were incubated with their cognate dye (i.e. DFHBI-1T or TO1-Biotin) for an hour at room temperature prior to being encapsulated into an emulsion later loaded into a glass capillary and imaged. Each com-

plex was exposed to a constant illumination their maximum excitation wavelength (i.e. 470 nm for Broccoli/DFHBI-1T and 508 nm for Mangos/TO1-Biotin) and their fluorescence emission was recorded respectively at 514 ± 24 nm and 540 ± 12 nm. All the values were background subtracted, relativized to the first measurement point and an exponential decay was fit to the data to compute the fluorescence half-life ($t_{1/2}$) of each complex (values indicated on the plot). A zoom on the first 10 seconds is shown in the inset. The values are the mean of three independent experiments and the error bars correspond to standard deviation. **(b)** Fluorescence stability of nucleoli signal in pSLQ-5S-F30-Mango IV expressing cells under constant illumination (dark orange) or pulsed excitation (200 ms, 0.2 Hz, light orange) with a 488 nm laser. Cells were fixed and stained with 200 nM TO1-Biotin. Constant illumination gave a $t_{1/2}$ = 11.7 ± 0.2 s whereas pulsed illumination significantly increases the lifetime >60 fold.

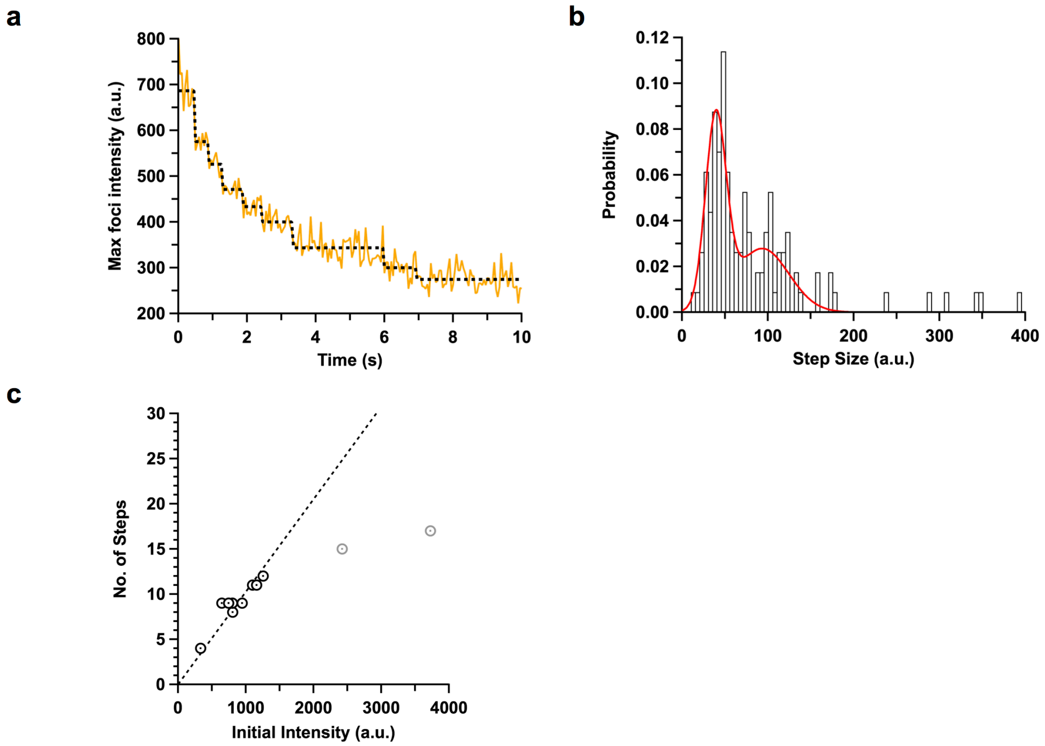

Supplementary Figure 19 | RNA Mango stoichiometry of individual foci (a) Representative fluorescent intensity trace (yellow) of a single 5S-F30-Mango IV foci from a fixed cell undergoing photobleaching under constant illumination (50 ms time resolution). Different intensity states (dashed line) are identified by maximum likelihood estimation, as described[3,2]. Between 4 and 17 photobleaching steps were observed for eleven different foci from fixed cells, indicating the number of bright 5S-F30-Mango IV molecules in each foci. **(b)** Distribution of photobleaching step sizes reveals two main peaks at ~40 and ~90 fluorescence intensity units, corresponding to photobleaching of one or two 5S-F30-Mango IV molecules. **(c)** Number of photobleaching steps per foci as a function of the initial intensity. Below 2,000 fluorescence units, the number of steps is proportional to the initial foci intensity (dashed line). Above 2,000 fluorescence units, the number of steps is under estimated due to multiple simultaneous photobleaching steps in the initial decay. Therefore, the grey points were excluded from the linear fit (dashed line).

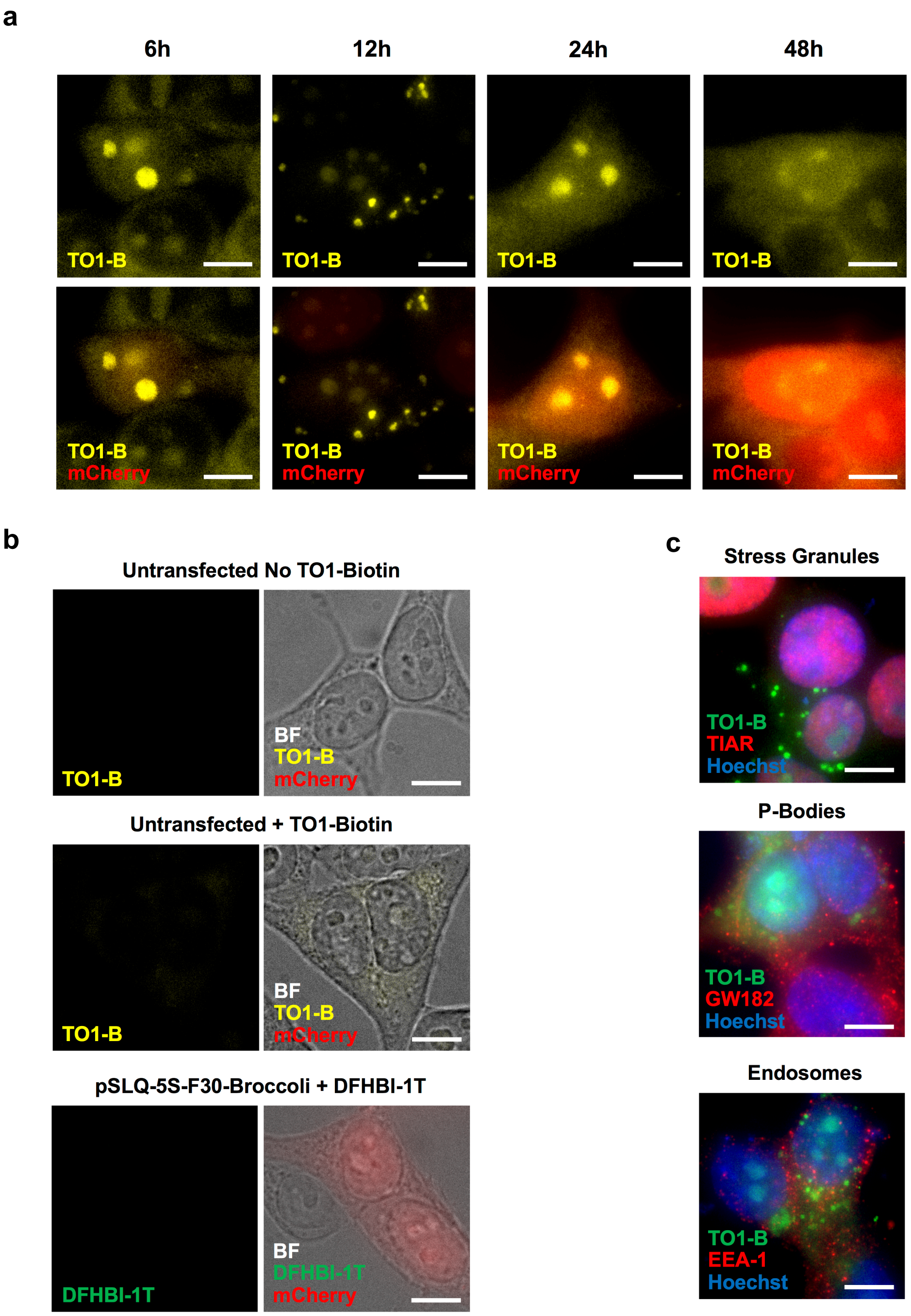

Supplementary Figure 20 | Imaging of genetically encoded aptamer tagged 5S rRNA. (a) Fixed time points of pSLQ-5S-F30-Mango IV expression, fixed and stained with 200 nM TO1-Biotin. TO1-Biotin – Yellow, mCherry – Red. **(b)** Fixed images of untransfected cells stained with 200 nM TO1-Biotin and pSLQ-5S-F30-Broccoli expressing cells stained with 10 µM DFHBI-1T. **(c)** Images of immunostained cells expressing 5S-F30-Mango IV and stained for stress granules (TIAR), P-bodies (GW182) and endosomes (EEA-1). TO1 signal – Green, Immunostain – Red and Hoechst 33258 (1 µg/ml) – Blue. All Scale bars = 10 µm.

Supplementary Table 1 – Metrics of the fluorescence profiling of Mango and R12

Round	Temperature (°C)	TO1-Biotin (nM)	λ value	Fusion efficiency (%)	Number of analyzed droplets	Number of analyzed variants
**R12-library**	25	100	1.25	95	168,441	200,023
**Mango**	25	100	0.54	95	63,200	1

Supplementary Table 2 – Metrics of screenings in presence of NMM

Round	Temperature (°C)	NMM (µM)	λ value	Fusion efficiency (%)	Number of ana-lyzed droplets	Number of ana-lyzed variants	Number of sorted droplets
**1**	45	3	1.25	90	2,716,500	3,056,062	25,834
**2**	45	4	0.20	85	1,011,000	171,870	21,754
**3**	45	6	0.22	87	1,511,250	289,253	3,719
**4**	45	8	0.14	75	986,875	103,621	5,309
**5**	45	0	0.15	90	491,625	66,369	1,689

Supplementary Table 3 – Metrics of screenings in the presence of TO3-Biotin

Round	Temperature (°C)	TO3-Biotin (nM)	TO1-Biotin (nM)	λ value	Fusion efficiency (%)	Number of ana-lyzed droplets	Number of ana-lyzed variants	Number of sort-ed droplets
**1**	45	0	100	1.25	95	2,328,630	2,765,248	4,607
**2**	45	3.5	100	0.20	80	1,106,750	177,080	8,328
**3**	45	5	100	0.15	94	1,019,990	143,818	6,446
**4**	45	7	100	0.20	95	1,002,500	190,475	11,478
**5**	45	15	100	0.17	90	1,044,500	159,808	12,205
**6**	45	30	100	0.25	85	1,015,880	215,874	8,779
**7**	25	60	100	0.20	79	1,087,500	195,750	8,463
**8**	45	115	100	0.26	88	768,250	175,775	4,729
**9**	25	230	100	0.28	75	547,250	114,922	5,868
**10**	45	0	25	0.15	70	499,500	52,447	4,004

Supplementary Table 4: Hill coefficients of RNA Mango/TO1-Biotin complexes

Mango	Hill Coefficient		K_D (mM)	
	K^+	Na^+	K^+	Na^+
**I**	1.2 ± 0.1	N/A	48 ± 5	N/D
**II**	1.4 ± 0.2	0.8 ± 0.1	39 ± 10	170 ± 110
**III**	1.0 ± 0.8*	0.5 ± 0.5*	0.4 ± 0.1	0.8 ± 0.3
**IV**	1.5 ± 0.2*	N/A	64 ± 24	N/D

Errors are the standard deviation of three independent measurements.
* Hill coefficients with an asterisk are estimated based on initial rise of fluorescence data.

Supplementary Table 5 – Constructs used in *in vivo* experiments

Construct	Sequence (5' → 3')
**5S-30-Control**	GUCUACGGCC AUACCACCCU GAACGCGCCC GAUCUCGUCU GAUCUCGGAA GCUAAGCAGG GUCGGGCCUG GUUAGUACUU GGAUGGGAGA CCGCCUGGGA AUACCGGGUG CUGUAGGCGU CGACUUGCCA UGUGUAUGUG GGGAAACCCA CAUACUCUGA UGAUCCUUCG GGAUCAUUCA UGGCAA
**5S-F30-Mango II mutant**	GUCUACGGCC AUACCACCCU GAACGCGCCC GAUCUCGUCU GAUCUCGGAA GCUAAGCAGG GUCGGGCCUG GUUAGUACUU GGAUGGGAGA CCGCCUGGGA AUACCGGGUG CUGUAGGCGU CGACUUGCCA UGUGUAUGUG GGUACGAAUU AGAUUAGAUU AAGAUUAGAG UACCCACAUA CUCUGAUGAU CCUUCGGGAU CAUUCAUGGC AA
**5S-F30-Mango I**	GUCUACGGCC AUACCACCCU GAACGCGCCC GAUCUCGUCU GAUCUCGGAA GCUAAGCAGG GUCGGGCCUG GUUAGUACUU GGAUGGGAGA CCGCCUGGGA AUACCGGGUG CUGUAGGCGU CGACUUGCCA UGUGUAUGUG GGUACGAAGG GACGGUGCGG AGAGGAGAGU ACCCACAUAC UCUGAUGAUC CUUCGGGAUC AUUCAUGGCA AUCUAGA
**5S-F30-Mango II**	GUCUACGGCC AUACCACCCU GAACGCGCCC GAUCUCGUCU GAUCUCGGAA GCUAAGCAGG GUCGGGCCUG GUUAGUACUU GGAUGGGAGA CCGCCUGGGA AUACCGGGUG CUGUAGGCGU CGACUUGCCA UGUGUAUGUG GGUACGAAGG AGAGGAGAGG AAGAGGAGAG UACCCACAUA CUCUGAUGAU CCUUCGGGAU CAUUCAUGGC AA
**5S-F30-Mango III**	GUCUACGGCC AUACCACCCU GAACGCGCCC GAUCUCGUCU GAUCUCGGAA GCUAAGCAGG GUCGGGCCUG GUUAGUACUU GGAUGGGAGA CCGCCUGGGA AUACCGGGUG CUGUAGGCGU CGACUUGCCA UGUGUAUGUG GGUACGAAGG AAGGAUUGGU AUGUGGUAUA UUCGUACCCA CAUACUCUGA UGAUCCUUCG GGAUCAUUCA UGGCAA
**5S-F30-Mango IV**	GUCUACGGCC AUACCACCCU GAACGCGCCC GAUCUCGUCU GAUCUCGGAA GCUAAGCAGG GUCGGGCCUG GUUAGUACUU GGAUGGGAGA CCGCCUGGGA AUACCGGGUG CUGUAGGCGU CGACUUGCCA UGUGUAUGUG GGUACCGAGG GAGUGGUGAG GAUGAGGCGA GUACCCACAU ACUCUGAUGA UCCUUCGGGA UCAUUCAUGG CAA
**5S Δ78-98-F30-Mango IV**	GUCUACGGCC AUACCACCCU GAACGCGCCC GAUCUCGUCU GAUCUCGGAA GCUAAGCAGG GUCGGGCCUG GUUAGUAGAA AGAAUACCGG GUGCUGUAGG CGUCGACUUG CCAUGUGUAU GUGGGUACCG AGGGAGUGGU GAGGAUGAGG CGAGUACCCA CAUACUCUGA UGAUCCUUCG GGAUCAUUCA UGGCAA
**U6-Control**	GUGCUCGCUU CGGCAGCACA UAUACUAAAA UUGGAACGAU ACAGAGAAGA UUAGCAUGGC CCCUGCGCAA GGAUGACACG CAAAUUCGUG AAGCGUUCCA UAUUUUU
**U6-Mango IV**	GUGCUCGCUU CGGCAGCACA UAUACUAAAA UUGGAACGAU ACAGAGAAGA UUAGCAUGGC CCCUACCGAG GGAGUGGUGA GGAUGAGGCG AGUAGGAUGA CACGCAAAUU CGUGAAGCGU UCCAUAUUUU U
**mgU2-47 Control**	AGCAAAGUGA UGAGUAAUAC UGGCUGGAGC CCCAAAGAGG CACGUGUGUG UGUUUGUGUG UGUGUGUAUA UGCUUGUCAG UGCAUGCACG UGUAUGUCUG GGAGUACAAA UGGGUGCGAC UGGUUGUAGG GAACUAGCUA UGUGCCUUCU AUUAGGCCAU GACAGUCAAA CUGAUAAGAU CUGAUUGCUU UUUUU
**mgU2-47 Mango II**	AGCAAAGUGA UGAGUAAUAC UGGCUGGAGC CCCAAAGAGG CACGUGUGUG UGUUUGUGUG UGUGUGUAUA UGCUUGUCAG UGCAUGCACG UGUAGGCACG UACGAAGGAG AGGAGAGGAA GAGGAGAGUA CGUGCCUAGG GAACUAGCUA UGUGCCUUCU AUUAGGCCAU GACAGUCAAA CUGAUAAGAU CUGAUUGCUU UUUUU

Supplementary Table 6 – Primers used for the construction of plasmids

Primer	Sequence (5' → 3')
**5' SalI F30 primer**	GGCGTCGACT TGCCATGTGT ATGTGGGTAC
**3' XbaI F30 primer**	CGCTCTAGAT TGCCATGAAT GATCCCGAAG G
**5' BamHI T7-5S primer**	GCCGGATCCT AATACGACTC ACTATAGTCT ACGGCCATAC CACCC
**5' BstXI 5S primer**	TTGGAGAACC ACCTTGTTGG GTCTACGGCC ATACCACCC
**3' XhoI F30 Rev primer**	GTACTCGAGA AAAAAATTGC CATGAATGAT CCCGAAGG
**5' BstXI mgU2-47**	TTGGAGAACC ACCTTGTTGG AGCAAAGTGA TGAGTAATAC TGGCTG
**3' XhoI mgU2-47**	GTACTCGAGA AAAAAAGCAA TCAGATCTTA TCAGTTTGAC TG

Supplementary References

1. Jeng, S. C. Y., Chan, H. H. Y., Booy, E. P., McKenna, S. A. & Unrau, P. J. Fluorophore ligand binding and complex stabilization of the RNA Mango and RNA Spinach aptamers. *RNA* **22,** 1884–1892 (2016).
2. Liao, Y., Li, Y., Schroeder, J. W., Simmons, L. A. & Biteen, J. S. Single-Molecule DNA Polymerase Dynamics at a Bacterial Replisome in Live Cells. *Biophys. J.* (2016).
3. Watkins, L. P. & Yang, H. Detection of intensity change points in time-resolved single-molecule measurements. *J. Phys. Chem. B* **109,** 617–628 (2005).
