# Supplementary material for: Fluorogenic RNA Mango aptamers for imaging small non-coding RNAs in mammalian cells
Source: Nat Commun. 2018 Feb 13;9:656. doi: 10.1038/s41467-018-02993-8 (PMC5811451; doi:10.1038/s41467-018-02993-8)
Supplement: Supplementary file 3 — Description of Additional Supplementary Files [file 41467_2018_2993_MOESM3_ESM.pdf]

Description of Additional Supplementary Files

File Name: Supplementary Movie 1
Description: **5S-F30-Mango III foci dynamics in live cells.** Dynamics of two adjacent 5S-F30-Mango III foci in live cells after direct transfection of RNA in complex with TO1-Biotin at a 2:1 molar ratio of dye to RNA. Stills of the movie can be seen in Supplementary Figure 14b. TO1-Biotin signal (Yellow) and EBFP2-H2B (Blue). The frame rate = 200 ms and the movie plays at 5 frames per second. Scale bars = 10 µm

File Name: Supplementary Movie 2
Description: **5S-F30-Control RNA transfection shows low background in live cells.** Background signal from 5S-F30-Control RNA after direct transfection in complex with TO1-Biotin at a 2:1 molar ratio of dye to RNA. TO1-Biotin signal (Yellow) and EBFP2-H2B (Blue). The frame rate = 200 ms and the movie plays at 5 frames per second. Scale bars = 10 µm

File Name: Supplementary Movie 3
Description: **Variation of 5S-F30-Mango III foci dynamics in live cells.** Dynamics of 5S-F30-Mango III across multiple cells shows three distinct diffusive behaviours. Static foci (Red track), slow localized diffusion (Blue track) and fast directed diffusion (Green track). This data is also plotted as RMSD is Supplementary Figure 14c. RNA was directly transfected in complex with TO1-Biotin at a 2:1 molar ratio of dye to RNA. TO1-Biotin signal (Yellow) and EBFP2-H2B (Blue). The frame rate = 200 ms and the movie plays at 5 frames per second. Scale bars = 10 µm
